# Public relations in health and medicine: using publicity and other unpaid promotional methods to engage audiences

**DOI:** 10.1186/s12913-020-05602-x

**Published:** 2020-09-15

**Authors:** James K. Elrod, John L. Fortenberry

**Affiliations:** 1 Willis-Knighton Health System, 2600 Greenwood Road, Shreveport, LA 71103 USA; 2 LSU Shreveport, 1 University Place, Shreveport, LA 71115 USA

**Keywords:** Public relations, Marketing communications, Promotion, Hospitals, Healthcare

## Abstract

**Background:**

Public relations—a marketing communications method involving the use of publicity and other unpaid promotional methods to deliver messages—historically has served as the communicative workhorse of the health services industry, representing the predominant pathway over many decades by which health and medical facilities conveyed stories to the public. While other components of the marketing communications mix, perhaps most notably that of advertising, have now captured a significant portion of interest, attention, and use by healthcare establishments, public relations remains a valuable communicative avenue when deployed properly.

**Discussion:**

As an unpaid method of promotion, public relations is uniquely positioned among its counterparts in the marketing communications mix which require direct expenditures to reach audiences. Typically effected by preparing and submitting press releases to news media firms in hopes that they, in turn, will present given stories to their audiences, limitations are somewhat obvious as transmission control rests with external entities. But overcoming limitations is possible with prudent strategies. This article presents Willis-Knighton Health System’s associated strategies, along with a range of public relations insights from decades of deployment experience.

**Conclusions:**

Prudently deployed and led by guiding strategies, public relations offers health and medical organizations opportunities to engage audiences in an efficient and highly credible manner. Courtesy of its unique properties, public relations capably can complement other marketing communications, operating synergistically to help healthcare institutions achieve their conveyance goals, fostering exchange and bolstering market share. Careful operationalization of this marketing communications avenue can help healthcare establishments realize their full communicative potential.

## Background

Few institutions possess greater needs to communicate than those which offer health and medical services to the public. Hospitals, medical clinics, and myriad other healthcare providers offer services of extreme importance to the populace, with these offerings affording individuals with opportunities to witness improved health and well-being, ultimately influencing broad community health. Life-enhancing, life-extending, and life-saving interventions are among the most important offerings provided in society [1, 2], but without proper conveyance of the availability of these services, audiences will remain uninformed and thus unable to take advantage of them. As such, poor or nonexistent communication on the part of healthcare establishments has the very real potential to diminish both individual and community health. It also has the potential to erode the financial viability of given healthcare establishments, as communication shortfalls will diminish opportunities to attract audiences who supply the all-important patronage that permits establishments to remain operational [3–11]. All in all, communication should not be taken lightly by healthcare providers, with success on this front being an essential ingredient for successful medical pursuits.

One method for communicating with current and prospective patients is through what is known as public relations. Public relations, one of five conveyance methods constituting the marketing communications mix, involves the use of publicity and other unpaid forms of communication to disseminate messages to desired audiences [4, 7, 12]. Public relations traditionally served as the primary method by which healthcare providers informed audiences of available medical services, accolades and awards, new technologies offered, and so on. It now, however, is complemented by the full range of the marketing communications mix, which also includes personal selling, sales promotion, direct marketing, and perhaps most notably, advertising [13]. Despite it being joined by other components of the marketing communications mix, public relations remains a valuable tool for the promotion of healthcare services [7, 8].

Effecting public relations proficiently in health services organizations requires devoted study and practical experience, permitting a current, expanding knowledge base which facilitates continual improvement. Additional benefits are afforded by studying the public relations strategies and tactics of peer health and medical providers, offering exposure to external approaches which might be adopted to enhance and improve communicative skills and abilities. This particular article shares public relations insights from Willis-Knighton Health System, shoring up experiential accounts available in the literature and providing food for thought for healthcare establishments seeking to bolster their public relations acumen.

## Definition and overview

Public relations falls under the broad discipline of marketing, formally defined as “a management process that involves the assessment of customer wants and needs, and the performance of all activities associated with the development, pricing, provision, and promotion of product solutions that satisfy those wants and needs” [7], p. 288. Promotion, as evidenced in this definition, is a core feature of marketing, earning inclusion as one of the Ps in the classic expression known as the *four Ps of marketing* (i.e., Product, Price, Place, Promotion). The promotion aspect of marketing essentially entails any and all elements associated with engaging audiences, with the core pathways for engagement being depicted in a descriptive model known as the marketing communications (or promotions) mix [7, 14].

Classically illustrated, the marketing communications mix contains five principal avenues of communication; namely, advertising (i.e., the paid use of mass media to deliver messages), personal selling (i.e., the use of sales agents to personally deliver messages), sales promotion (i.e., the use of incentives, such as contests and free giveaways, to encourage patronage), public relations (i.e., the use of publicity and other unpaid promotional methods to deliver messages), and direct marketing (i.e., the delivery of messages via mail, the Internet, and similar routes directly to consumers) [7, 8]. Health and medical providers consider each of these communicative avenues, selecting one or more deemed to be best suited for reaching target audiences, with the ultimate goal being to encourage patronage or prompt some other desired action [7, 15].

The reliance of public relations on unpaid methods of promotion positions it uniquely among the components of the marketing communications mix, as other pathways require direct expenditures to effect audience engagement. The typical manner in which healthcare institutions effect public relations is by preparing and forwarding press releases profiling noteworthy matters to news media outlets in hopes that they, in turn, will disseminate given stories to their audiences. The media firms carrying associated stories do not charge a fee for such, as the items are deemed to be of interest to their audiences. While the cost-free aspect of public relations is highly attractive, the downside is that it places healthcare institutions in positions of reliance on outside parties—news media organizations—to accept and present forwarded stories. This, of course, offers no guarantees of circulation, as the stories can be rejected for any number of reasons, including perceived audience disinterest, limited allotments of space or time for presentation, breaking news stories which crowd out content deemed to be of less importance, and so on. Even if carried, intended stories may not be communicated as desired, as editorial control rests with conveying news media firms. Regardless of such, especially when used in tandem with other components of the marketing communications mix which do offer guarantees of message dissemination, public relations can provide useful communications utility for healthcare organizations [13, 14, 16].

Public relations historically has served as the communicative workhorse of the health services industry, representing the predominant pathway over many decades by which health and medical facilities conveyed stories to the public. In fact, it generally afforded the only way for health services institutions to gain a mass media presence up to the 1980s. Prior to the 1980s, advertising—at that time, a well-established communicative method relying on paid use of mass media and a staple of promotion outside of the health services realm—was shunned as being beneath the dignity of health and medicine. Advertising also was feared for its potential to upset patient referral patterns and, notably, the American Medical Association banned its use by members [4, 6, 7, 12, 13]. Such influences and traditions ultimately compelled most health services providers to find other avenues for engaging consumers, with public relations being the operative method of the era for achieving a mass media presence [13, 14]. Resistance to advertising ceased in the 1980s, helped by the American Medical Association’s relinquishment of its ban on members’ use of advertising following US Federal Trade Commission scrutiny, and today it thrives, affording health services providers with unfettered access to mass media channels [4, 6, 7, 12, 13]. Still, the formative years of reliance on public relations made a lasting impression which continues to influence health services communications to this day.

## Institutional background and deployment history

Established in 1924, Willis-Knighton Health System possesses a rich history of successfully communicating with current and prospective patients. Communications excellence, in fact, has been and continues to be viewed as a strategic priority, compelling extensive use, experimentation, and innovation. Based in Shreveport, Louisiana and situated in the heart of an area known as the Ark-La-Tex where the states of Arkansas, Louisiana, and Texas converge, Willis-Knighton Health System holds market leadership in its served region where it delivers comprehensive health and wellness services through multiple hospitals, numerous general and specialty medical clinics, an all-inclusive retirement community, and more. The achievement of market leadership can be credited, in part, to communications prowess, permitting the institution to effectively engage customer groups, yielding significant interest and attention, ultimately leading to all-important patronage and customer loyalty.

Today, Willis-Knighton Health System leverages the power of the full marketing communications mix, but there was a time period in which it primarily turned to public relations for engaging audiences, following industry traditions, as described in the prior section. This proved sufficient for much of Willis-Knighton Health System’s early history. In this era, the institution focused primarily on serving the population of west Shreveport and found that its communications goals were largely met by relying on news media outlets to present submitted accounts to their respective audiences. However, in the 1970s, Willis-Knighton Health System sought to grow its service area beyond west Shreveport, desiring an expanded geographic presence. To achieve this bold vision, the institution pursued a range of growth-fueling innovations, including adoption of the hub-and-spoke model [17, 18], establishing centers of excellence [19], expanding physical space via the practice of adaptive reuse [20, 21], and more. Knowing that these new initiatives would count for very little without attracting increasing numbers of patients, Willis-Knighton Health System carefully assessed its communications efforts to ascertain their potential for delivering the utility necessary to meet its growth ambitions.

In evaluating its lengthy history of reliance on public relations initiatives, Willis-Knighton Health System believed that, if used in isolation, this particular avenue would not deliver sufficient communications power to achieve desired goals. The institution had experienced firsthand the frustration of forwarded press releases not being published by news media outlets, confirming that sole reliance on this pathway, especially for critical communications, would be problematic [13, 16]. As such, Willis-Knighton Health System decided that complementary communications would be necessary, with this prompting the institution’s embracement of advertising, making it one of the earliest health services providers to do so, going against the traditions of the day [9, 13, 14, 16]. Still, this did not compel Willis-Knighton Health System to forgo pursuing public relations communications avenues. Instead, it continued its historic practice of preparing and submitting press releases to media outlets. If press releases were accepted for dissemination to audiences, the institution considered it to be a bonus; a cost-free communications gain. If press releases were rejected, it mattered not, as other deployed communications avenues, most notably, advertising, would ensure that conveyance goals were met. This particular approach to public relations proved to be effective and enduring [13].

## Context within marketing communications

**Fig. 1:**
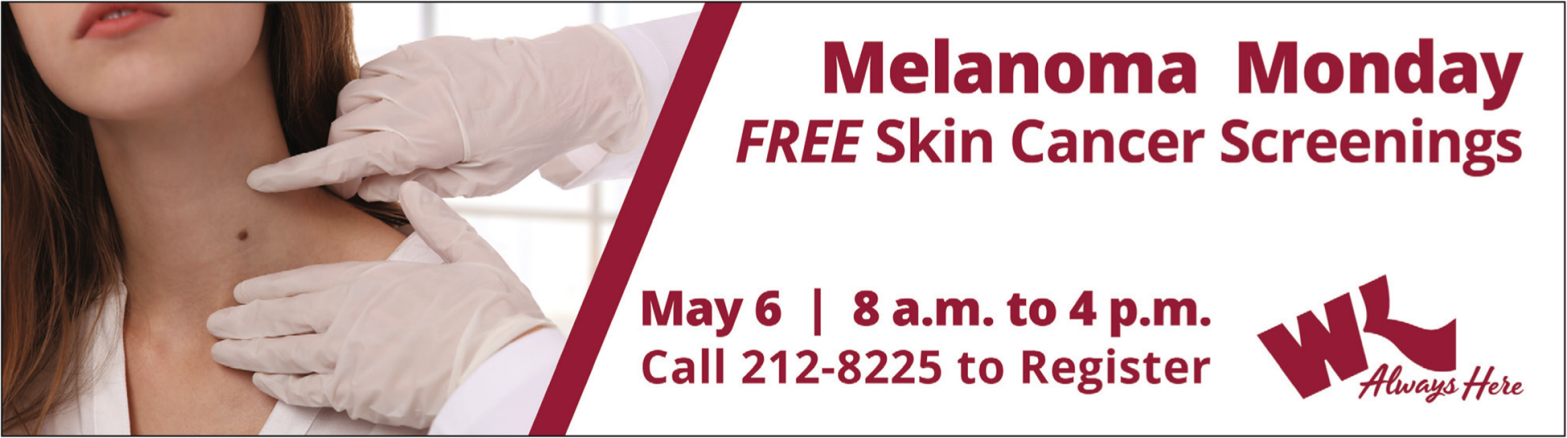
A billboard advertisement, supporting public relations communications, announcing the availability of free skin cancer screenings at Willis-Knighton Health System

In present day, decades after devising its formative marketing communications strategy in the 1970s, Willis-Knighton Health System’s approach to conveyances remains much the same, with the institution continuing to fully deploy the marketing communications mix, strategically selecting communications pathways to achieve its given aims. Regarding public relations, specifically, Willis-Knighton Health System’s approach also remains parallel to that established decades ago. The institution prepares and submits press releases to news media organizations, but relies on other forms of communication, most notably, advertising, for informing audiences of the associated events and opportunities [13, 14, 16]. Figure 1 presents a recent billboard advertisement supporting associated public relations communications, demonstrating this approach.

While the institution’s public relations approach remains the same, the stature and scale of Willis-Knighton Health System is vastly different than it was on forming its associated operational philosophy. The fantastic growth experienced by the institution over the past several decades has led to a dramatic increase in the variety and frequency of publicity-generating activities and events benefiting individuals and the greater community. Open houses, informative seminars, health fairs, technology acquisitions, and the like are routine across the system, motivating communicative efforts to inform the public of such, permitting improved health and wellness. But despite consistent distribution of press releases, just as was the case in the 1970s, Willis-Knighton Health System continues to view the number of publication acceptances and, of those accepted, the quality of presentation, to be unsatisfactory. This, of course, validates in present day the institution’s operational approach which treats public relations successes as welcomed bonuses, but relies on other components of the marketing communications mix to deliver desired conveyances [13].

## Strengths

Willis-Knighton Health System has turned to public relations for the duration of its existence, finding this conveyance method to be a worthy communicative mechanism when deployed properly. Several facets associated with public relations have motivated its continued use in efforts to engage current and prospective patient populations. These strengths are described as follows.

### Cost efficiency

Deploying public relations as a marketing communications device is perhaps motivated most intensely by its cost efficiency. As it relies on the unpaid use of mass media, healthcare establishments are not burdened with costs associated with buying air time, space, or any other associated media expenditure. The newsworthy nature of public relations conveyances effectively gives news media firms the ability to grant these presentations free of charge. Depending on the media source and the size of its audience, such allotments can be extremely expensive, representing a major cost savings for healthcare institutions which successfully secure coverage through public relations [7, 8, 22–26].

Of course, in many contexts, public relations is not exactly free, as the activities and events which make items newsworthy (e.g., free medical screenings, presentations by medical experts) carry costs associated with their provision. Without coinciding mass media expenditures, however, the cost factor is diminished considerably. Of course, cost-free avenues are possible, depending on the nature of the newsworthy matter. Press releases announcing staff member accomplishments, for example, are virtually cost free, as such accolades typically are pursued without public relations intentions and do not require additional investments (e.g., hosting an event). Public relations efforts of this sort when carried by news media outlets certainly could be considered to be free of any meaningful cost associated with the given conveyances. Regardless of whether cost free or merely cost efficient, public relations offers significant value [7, 8, 22–26].

### High credibility

Since public relations announcements successfully accepted for publication by news media organizations are ultimately conveyed as news pieces, audiences naturally view them to be highly credible, with the given stories being elevated by the reputations of the presenting news entities. Many consumers are skeptical of commercial conveyances, such as advertising and direct mail, as these are funded by the promoting parties. However, when activities, events, accomplishments, and the like are presented by news media firms, skepticism diminishes, positively influencing audience receptivity and potentially generating greater attention and awareness than that afforded by paid marketing communications applications [7, 8, 22–26].

The credibility factor associated with public relations carries significant weight, especially when health services organizations follow Willis-Knighton Health System’s formula for deployment. To illustrate a routine scenario, assume that a particular award for excellence was achieved by the institution. Strategy associated with communicating this accomplishment would be formulated via discussions between Willis-Knighton Health System’s Office of the President and the Department of Marketing and Public Relations. Typically, receipt of a prominent award would call for public relations, advertising, and direct marketing (notably including direct mail and social media). The institution also would promote the accolade via other promotional channels, such as digital billboards owned and operated by the system. Assuming that news media organizations decide to carry the associated story, the credibility afforded by such can be leveraged further by the paid communications methods which are deployed, offering synergies which amplify and reinforce the news announcements. As such, it is advised that health services organizations seek to use public relations in tandem with other communications avenues whenever opportunities for such emerge, as doing so can tap into the spillover effects generated by the credibility of associated news stories.

### Narrative guidance

Public relations is especially effective at guiding health services organizations in developing and advancing narratives which present efforts benefiting given communities. In order to attract and secure media coverage as newsworthy occurrences, submissions must indeed supply information that is of interest and benefit to audience members [7, 13]. Simply presenting an advertisement as a news story is unacceptable and almost certainly will result in an automatic rejection by news media outlets. This newsworthy mandate forces healthcare providers to view their activities and accomplishments from the perspective of stakeholders in the marketplace, notably ascertaining how the item or items supplied in accounts will positively impact individuals and communities. This also is a good test as to whether the given story is acceptable for public relations conveyances or better suited for other methods of communication.

Stories which are unacceptable should not be submitted as newsworthy events, as doing so can erode the credibility of healthcare institutions, diminishing chances of acceptance and circulation of stories which actually do have value to audiences. However, those stories which indeed are newsworthy certainly should be directed through public relations channels, accordingly. Many accomplishments and initiatives offered by healthcare providers afford significant community benefit, but sometimes institutions neglect to present these as such, opting for an overly commercial tone. A public relations mindset can be immensely helpful in framing stories, ensuring that opportunities to express accounts of benefit to the community are not inadvertently overlooked.

## Limitations

Despite compelling motivations for use, public relations as a communicative pathway must be treated carefully, as it possesses several key limitations. With knowledge of each, however, health services organizations can structure communications in a manner to benefit from the avenue’s strengths without being impeded by its limitations. Notable limitations are described as follows.

### Transmission uncertainty

Healthcare establishments considering public relations must always remember that the particular communications route offers no guarantees that events, activities, and other stories desired for conveyance will actually reach audiences, as such decisions rest with the news media organizations that oversee given communications platforms. Ultimately, healthcare organizations have no control over when or even if the information they supply will be transmitted to audiences. This lack of reliability quite obviously requires public relations to be treated in a particular manner, engaging in its pursuit while placing reliance on other components of the marketing communications mix to ensure circulation of information to desired groups [13, 16].

Beyond such a strategy, health services organizations can and should seek to build relationships with given news media organizations. It is very common for communities to experience health-related events which prompt journalists to investigate and report on such matters (e.g., an especially bad flu season and its impact on the populace). Very often, in developing stories, reporters will contact healthcare institutions, seeking feedback from medical experts. When requests can be accommodated, they can help build bridges of communication with news media firms and this, in turn, can be helpful in realizing greater attentiveness to submitted press releases. This is where a formal public relations office can prove to be a valuable asset, serving as the communications liaison between the establishment and external entities seeking information on any number of topics or concerns, fielding inquiries and connecting parties when prudent to do so. While building relationships with news media firms can be helpful, it is not a foolproof solution, as space and time limitations at least occasionally will prohibit circulation of even the most compelling accounts. Still, such efforts can improve the odds of acceptance and circulation of associated stories.

### Content presentation uncertainty

Closely related to public relations transmission uncertainty is that of content presentation uncertainty. Even if press releases are accepted for publication, the manner of presentation selected by given news media firms may not be as desired, as editorial control rests with the presenting organizations. Occasionally, press releases are conveyed exactly as submitted, but typically modifications of some sort will be effected. These modifications often entail minor alterations (e.g., content reductions), but occasionally they can be quite extensive, so much so that, in some cases, meaning is actually distorted [13, 16]. Overcoming this challenge is difficult, but it can be minimized by studying the stories presented by targeted news outlets, ensuring that efforts are made to provide content in submitted press releases which conforms with the style and presentation observed. This reduces editing burdens on the part of news firms, potentially hastening direct, accurate, and complete circulation of content.

### Return on investment uncertainty

Events, activities, and other pursuits which rely solely on public relations are risky, as failed coverage can harm return on investment [13, 16]. Assume that a health services provider invites a nationally-recognized expert on cardiovascular health to speak at an event open to the public which details the latest insights on heart health and wellness. Assume also that the event is held to (1) educate the public on heart care and (2) drive business to the institution’s cardiovascular unit. If public relations efforts (assuming their use in isolation) fail to secure media coverage (which is a very real possibility), audience size very likely will be reduced due to lack of awareness of the event. A diminished audience naturally will yield a diminished potential for the institution to witness an acceptable return on investment as admission and referral opportunities will be lessened. This particular concern, of course, can be moderated by deploying tandem communications alongside public relations efforts. This further supports the need for healthcare institutions to devise a formal marketing communications strategy which outlines how each and every component of the marketing communications mix, including public relations, is to be deployed, playing to each element’s strengths while minimizing or avoiding associated limitations.

## Operational reflections

For administering any component of the marketing communications mix, Willis-Knighton Health System advises establishing a baseline foundation of resources, including (1) top leadership support and commitment, (2) financial resources sufficient for funding communications activities, (3) competent personnel charged with effecting given initiatives, and (4) formal processes permitting effective planning, implementation, and evaluation of initiatives. Adequate resources set the stage for productive audience engagement endeavors, minimizing chances of resource-depleting and reputation-damaging mistakes which, in the realm of marketing communications, often are very public, given the open circulation of such conveyances. These resources also ensure competencies in using given marketing communications mix components, with proper deployment being essential for realizing desired outcomes.

As for public relations, specifically, beyond the advisories conveyed elsewhere in this article, Willis-Knighton Health System recommends that healthcare institutions consider establishing formal public relations departments. Serving as the liaison between an organization and external parties, such departments add a beneficial resource [22–26]. As noted earlier in this article, community stakeholders and other publics often seek information from healthcare organizations on a range of topics. In such cases, a public relations department can field inquiries, address them directly if permissible, or route them as needed to others to handle. This resource proves helpful not only in positive circumstances, but also in cases of negative publicity requiring damage control measures. Further, the experts housed in these departments can elevate the quality of press releases issued by healthcare establishments. Notably, they can ensure that stories are newsworthy, titled in a manner to attract attention, grammatically correct, styled in a fashion consistent with that of stories carried by targeted outlets, and presented concisely to facilitate screening, potentially aiding in garnering acceptances. Dedicated personnel can also direct time toward building relationships with news media organizations, with this, too, fostering circulation.

Structurally, based on the desires of healthcare establishments, the public relations function can be organized as an independent unit or placed within an existing marketing department. Regardless of structural arrangement, healthcare establishments should clearly identify responsible units as public relations components, permitting members of the press, residents of the community, and other inquirers to quickly locate those who can address their questions and concerns. All in all, healthcare providers would do well when devising their marketing communications strategies to consider expanding the public relations component, permitting realization of its full potential.

## Conclusions

As Willis-Knighton Health System has experienced, when prudently deployed and led by guiding strategies, public relations offers health and medical organizations opportunities to engage audiences in an efficient and highly credible manner. Courtesy of its unique properties, public relations capably can complement other marketing communications, operating synergistically to help healthcare institutions achieve their conveyance goals, fostering exchange and bolstering market share. Extending the role of public relations to incorporate duties as liaison between institution and community also adds significant benefits which can assist healthcare establishments in achieving comprehensive communications. Careful operationalization of this marketing communications avenue can help healthcare institutions realize their full communicative potential.

## Data Availability

Not applicable.
